# RETURN TO WORK AFTER CARPAL TUNNEL RELEASE SURGERY

**DOI:** 10.1590/1413-785220253303e292278

**Published:** 2025-12-01

**Authors:** Renata Gabriela Pereira Cunha Pontes, Anderson Clayton Cardeal, Mariana Avelino dos Santos, Luís Guilherme Rosifini Alves Rezende, Nilton Mazzer, Edgard Eduard Engel

**Affiliations:** 1 Universidade de Sao Paulo, Faculdade de Medicina de Ribeirao Preto, Sao Paulo, SP, Brazil.

**Keywords:** Return to Work, Carpal Tunnel Syndrome, Social Security, Retorno ao Trabalho, Síndrome do Túnel do Carpo, Previdência Social

## Abstract

**Objective:**

To clarify whether work-related factors influence the return to work after CTS surgery.

**Methods:**

Descriptive observational study that included 56 patients who underwent CTS surgery. The variables studied were profession, employment status, time off work after surgery, reason for returning to work, stress level, leave granted by the National Institute of Social Security (INSS), change in job function, and level of work effort.

**Results:**

The average time to return to work was 39.8 days (SD: 22.3). Self-employed individuals returned to work 6.5 days (SD: 22.3) earlier than salaried employees with a formal contract, who returned in 43.8 days (SD: 23.9; p=0.49). Most patients were on leave granted by the INSS, with an average of 47.8 days (SD: 23; p=0.003). Patients who had surgery on their left upper limb returned to work one week earlier than those who had surgery on their right (p=0.025).

**Conclusion:**

Coverage by INSS are associated with a delay of approximately two weeks in return to work compared to patients without this coverage. *Level of Evidence II; Retrospective* ^f^ *Study.*

## RESUMO

**Objetivo:**

Esclarecer se fatores relacionados ao trabalho influenciam o retorno ao trabalho após cirurgia para síndrome do túnel do carpo (STC).

**Métodos:**

Estudo observacional descritivo que incluiu 56 pacientes submetidos à cirurgia para STC. As variáveis estudadas foram profissão, situação empregatícia, tempo de afastamento do trabalho após a cirurgia, motivo para o retorno, nível de estresse, licença concedida pelo Instituto Nacional do Seguro Social (INSS), mudança na função ocupacional e nível de esforço no trabalho.

**Resultados:**

O tempo médio para retorno ao trabalho foi de 39,8 dias (DP: 22,3). Trabalhadores autônomos retornaram ao trabalho 6,5 dias (DP: 22,3) antes dos empregados formais, que retornaram em 43,8 dias (DP: 23,9; p=0,49). A maioria dos pacientes estava de licença concedida pelo INSS, com uma média de 47,8 dias (DP: 23; p=0,003). Pacientes que realizaram a cirurgia no membro superior esquerdo retornaram ao trabalho uma semana antes daqueles operados no membro direito (p=0,025).

**Conclusão:**

Pacientes com cobertura pelo INSS estão associados a um atraso de aproximadamente duas semanas no retorno ao trabalho em comparação com pacientes sem essa cobertura. *Nível de Evidência II; Estudo Retrospectivo* ^f^.

## INTRODUCTION

Carpal Tunnel Syndrome (CTS) is the most common compressive syndrome in the upper limb, characterized by the compression of the median nerve in the carpal tunnel.^1^ In severe cases, motor function and grip strength are affected, leading to work absenteeism and reduced productivity. Over the past 5 years in Brazil, 2,855 sick leaves and 1,081 retirements were granted for individuals with CTS.^2^

CTS is widely associated with occupations involving repetitive movements and extreme wrist positioning, being common among manual Workers.^3^ Surgery has reported success rates between 71% and 90%,^4,5^ and the lack of clear guidelines to guide return to work after CTS surgery represents a significant challenge, as the length of absenteeism can have a substantial impact on the economy and patient well-being. A British study revealed that return-to-work guidelines after CTS surgery vary considerably depending on the type of work performed, creating inconsistencies in postoperative management.^6^

The return to work of Brazilian patients has an important particularity: the influence of the National Institute of Social Security (INSS). However, the relationship between these factors has not yet been explored in the literature. This study aims to identify the factors related to the time of return to work after surgical treatment of CTS in Brazilian patients, considering the particularities of the social security system, and thus fill a gap in the medical literature, providing information to help surgeons guide a safe return to work.

## MATERIALS AND METHODS

Descriptive observational study using electronic medical record data complemented by online questionnaires completed by patients. The study population included patients who underwent CTS surgery at the Hospital das Clínicas of the Ribeirão Preto Medical School, University of São Paulo, and at the State Hospital of Serrana from January 2015 to October 2022.

Employed patients at the time of surgery were included. Responses from patients who did not properly sign the Informed Consent Form (ICF) were excluded. Medical record data included epidemiological information (age, gender, ethnicity, comorbidities), disease-related data (laterality, clinical examination results at the first evaluation, severity of electromyography at diagnosis, presence of thenar atrophy), and surgical information (postoperative complications).

Data not available in the medical records was collected through a questionnaire sent via WhatsApp® to all patients who met the inclusion criteria. The variables collected through the questionnaire included: type of job, employment relationship, time away from work after surgery, cause of early or late return (open response), perceived stress level at work, perceived stress level at home, receipt of INSS leave, and need for job change. Patients also characterized their job as light or heavy manual work.

The questionnaire presented open responses.

The research project and instrument were approved by the Research Ethics Committee of HCFMRP-USP under CAEE: 45539421.3.0000.5440.

**Figure 1:**
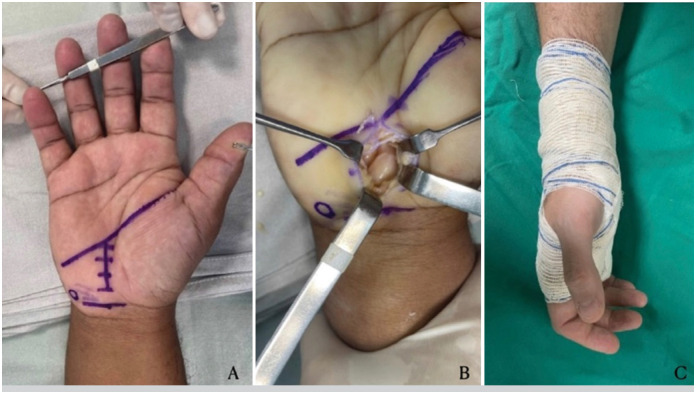
(A) Surgical planning, highlighting the incision site. (B) Identification of the nerve post-neurolysis. (C) Application of a soft splint for postoperative support.

Source: Photo archive of the Hand Surgery Residence at FMRP-USP.

All patients underwent the same surgical technique, involving open carpal tunnel release through a mini-incision under local anesthesia and sedation. Complete release of the transverse carpal ligament was performed. Patients were discharged on the same day of surgery with a soft splint. (Figure 1).

In this study, of the 445 surgeries performed, several groups were excluded from receiving the questionnaire: 6 patients had passed away, 4 were unemployed, 71 were retired, and 108 were housewives. Consequently, the questionnaire was sent to 256 patients, resulting in 75 responses. Further exclusions were made based on incomplete or incorrect data: 4 patients were excluded due to questionnaire errors, 7 had not yet returned to work, 2 were waiting for surgery on the contralateral side, and 6 housewives had incorrect information on record. As a result, 56 patients were deemed eligible for analysis.

## RESULTS

Most patients were female (87.9%), with 69.6% being white, 21.5% mixed-race, and 8.9% black (Table 1). This racial distribution was consistent with the general population according to the 2010 census. The average age was 44 years, ranging from 27 to 64 years, following the international trend of CTS affecting primarily individuals between 40 and 60 years old.^3,7,8^

**Table 1:** Epidemiological variables and return to work.

Variable	Mean return to work (days)	SD	p-value
**Self-declared race**			
White	36.5	23.4	0.1
Mixed	51.3	19.6	
Black	39.8	15.1	
**Gender**			
Female	40.7	22.5	0.8
Male	37.4	25.5	

Regarding the affected hand, 48.2% of patients presented with symptoms in the right hand, 23.2% in the left hand, and 28.6% in both hands. The majority of patients (74%) had electromyography findings consistent with moderate to severe CTS, likely reflecting the fact that the study was conducted in specialized Hand Surgery referral centers, which generally receive more advanced cases due to the time required for referral processes. Additionally, part of the study period coincided with the COVID-19 pandemic, during which elective outpatient services were suspended, potentially delaying diagnosis and treatment.

The Phalen test was the most frequently positive (69.6%), followed by the Durkan test (64.2%) and Tinnel test (60%). None of the clinical examination data, including the presence of thenar atrophy, showed an association with return to work time.

**Figure 2:**
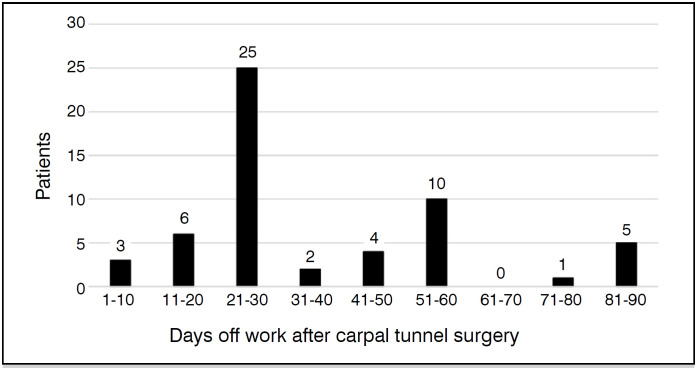
Patient distribution by time to return to work (in days).

The average return to work was 39.8 days, ranging from 3 to 90 days. More than half of the patients (55.4%) returned to work between 15 and 30 days after surgery (Figure 2). The main reasons for returning to work were financial necessity, reported by patients responsible for supporting their families, as well as the desire to work and perceived improvement after surgery (Table 2).

**Table 2:** Reason for return to work after CTS surgery.

Reasons given by patients for returning to work due to financial necessity	Reasons given by patients for returning to work due to pleasure in working or improvement in condition
"I have children to support."	"I like working."
"Single mother of three children."	"The desire to work."
"I need to work, I am alone, not retired."	"I love what I do."
"The severity of the problem and the pain it caused did not motivate me to return. I resigned from the company because the recovery would take many months and I would still have to operate on the other hand… However, I got another job where there is no heavy labor, and due to that, I have less difficulty and pain."	"I returned to work because I was doing well."
"Necessity, without work there is no money."	"The quick improvement."
"Financial problems… children to take care of, etc…."	"My hand was normal, and because I need to help at work."
"My bills are coming, and the only source of income I have is work."	"After the surgery, my hand was much better."

The perception of a successful surgical outcome was associated with an earlier return to work. In this group, the average time was shorter (36.3 days, SD: 20.7) compared to those who rated their hand as the same (47.5 days, SD: 27.5) or worse (55 days, SD: 22.9) than before surgery (p=0.08).

Regarding INSS leave, 57.1% of patients used this benefit, with an average of 47.8 days (SD: 23.8) compared to 29.1 days (SD: 14.8) for patients not on leave (p=0.003). The multivariate analysis corroborated the bivariate analysis data. Patients on INSS leave returned on average 16.7 days later than those not covered by the benefit, ranging from 5.9 to 27.4 days more (p=0.002).

Patients who considered their stress level high at home tended to return to work earlier, while those who reported high stress at work took longer to return (Tables 3 and 4).

**Table 3:** Impact of Home Stress Levels on Return-to-Work Time.

Stress level at home	N	%	Mean days to return to work	SD
Low	22	39.3%	43	27.5
Moderate	26	46.4%	38	18.6
High	8	14.3%	35	18.5

**Table 4:** Impact of Work Stress Levels on Return-to-Work Time.

Stress level at work	N	%	Mean days to return to work	SD
Low	10	17.9%	34	25.0
Moderate	21	37.5%	38	20.8
High	22	39.3%	42	21.8
No response	3	5.4%		

Regarding laterality, patients who underwent surgery on their non-dominant hand returned to work 2 weeks earlier than those with bilateral symptoms (p=0.025) (Table 5). Although we did not have sufficient data on hand dominance in the medical records for analysis, given that the global population is predominantly right-handed (90.7%),^9^ it is likely that patients who had surgery on their non-dominant hand experienced a quicker return to work.

**Table 5:** Multivariate analysis.

Variable	Unstandardized coefficient*	Confidence interval	p-value
**Race**			
White	-0.3	(-17.5; 16.8)	0.969
Mixed	5.7	(-14; 25.5)	0.570
Black (reference)			
**CTS laterality**			
Right CTS	-8.4	(-20.6; 3.7)	0.176
Center CTS	-15.6	(-29.2; −1.9)	0.025
Bilateral (reference)			
**Work perception**			
Light manual	-5.6	(-16.9; 5.6)	0.330
Heavy manual (reference)			
**INSS leave**			
Patient on leave	16.7	(5.9; 27.4)	0.002
Patient not on leave (reference)			
**Surgical outcome perception**			
Same as before surgery	9.2	(-6.3; 24.9)	0.245
Worse than before surgery	15.3	(-0.4; 31.1)	0.056
Better than before surgery (reference)			
**Hypertension (HTN)**			
Without HTN	10.5	(-0.06; 21.2)	0.051
With HTN (reference)			
**Phalen's test in the first consultation**			
Positive Phalen	3.9	(-7.2; 15.1)	0.489
Negative Phalen (reference)			
**Household tasks during the leave**			
Performed	-5.8	(-16; 4.2)	0.258
Did not perform (reference)			
**Employment relationship**			
Salaried with a formal contract	3.6	(-7.3; 14.6)	0.512
Salaried without a formal contract (reference)			

* Unstandardized coefficient: represents an average change compared to a standard value. Negative results: return to work before the reference. Positive results: return to work after the reference.

## DISCUSSION

The data revealed significant variability in return-to-work times, with some patients experiencing notably longer recovery periods than others. This variability underscores the influence of a wide range of factors, including individual patient characteristics and external factors such as access to INSS leave and social support, all of which play a crucial role in determining recovery time.

Traditional clinical factors, such as the presence of atrophy or results from specific clinical tests, did not show a significant association with delayed return to work. This suggests that purely clinical characteristics of CTS are not predictive of recovery time and return to work. A study with 50 workers in Israel found no association between positive clinical tests and longer return-to-work period.^10^

Patients who rated surgical outcomes positively tended to return to work earlier. This highlights the importance of communication between doctors and patients to set realistic expectations and build trust in the treatment. Dissatisfied patients with CTS surgery outcomes tend to either not return to work or return late, as described in a French cohort involving 935 patients.^11^

Financial needs of patients were identified as a motivation for returning to work. Many patients were primary breadwinners and faced economic pressures to resume work. This finding reinforces the influence of socioeconomic factors on the return-to-work process, previously described in the literature.^1^

Approximately 39.3% of patients returned to work after more than 30 days post-surgery, indicating a substantial period of absenteeism for a significant portion of the studied population. The average return-to-work period after CTS surgery was 39.8 days, with most patients returning between 15 and 30 days. Those who returned within 15 days generally performed jobs that did not require intense use of the hands. This 2-week earlier return for light manual workers was also found by De Kesel et al. in a study with 107 cases.^12^

The use of INSS benefits by patients was strongly associated with a delayed return to work, which highlights the direct influence of social security policies. On the other hand, patients who did not receive such benefits often had to return to work prematurely, even before full recovery. This dual scenario underscores the need for public policies that ensure financial support during postoperative recovery, without forcing early returns due to economic necessity.

The relationship between stress levels at work and delayed return underscores the importance of managing work stress as a potential target for interventions that can expedite return to work. In the short term (up to 60 days), patients with high psychological demand jobs are less likely to return to work successfully compared to those with low work stress levels.^13^

The main limitation of this study, which may have impacted the results, was the limited response rate (29%) to the questionnaire by patients, who reported fear of scams when clicking on links sent via WhatsApp. However, as the first Brazilian research studying return-to-work time after CTS surgery and the influence of INSS during this period, it offers important insights into patient recovery and the role of social security policies.

## CONCLUSION

This research showed that coverage by INSS is associated with a delay of approximately 2 weeks in return to work compared to patients without this coverage. When surgery is performed on the non-dominant hand, patients tend to return to work more quickly. Psychosocial factors, such as positive perception of surgical outcomes, low work stress, and high home stress, as well as economic factors, showed a tendency for earlier return to work.
